# Complete genome sequences of *Mycobacterium smegmatis* mc^2^155 cluster E bacteriophages Policronamos and Palpatine

**DOI:** 10.1128/mra.00392-25

**Published:** 2025-07-23

**Authors:** Shule M. Aggabao, Rebecca Hassell, Bryson S. Leary, Sheena I. Bautista, Hayzen H. Chamberlain, Kyla Radke, Harry M. Peless, Payson C. Danielson, Matthew N. Jackson, Hyunbi Hwang, Jacob D. Scott, Elisa A. Correa Lazaro, Atalie B. Bogh, Jayden S. Longhurst, Spencer T. Payne, Parker Danielson, Natalie A. Olsen, Christopher C. Harrell, Austin M. Johnson, Thomas Wilhite, Jeffrey K. Schachterle, Staci Avery, Donald P. Breakwell, Brett E. Pickett

**Affiliations:** 1 Department of Microbiology and Molecular Biology, Brigham Young University 723033, Provo, Utah, USA; Loyola University Chicago, Chicago, Illinois, USA

**Keywords:** phage, bacteriophage

## Abstract

Mycobacteriophages Policronamos and Palpatine are tailed phages infecting the *Mycobacterium smegmatis* mc^2^155 strain. The genome for Policronamos is 75,894 bp long with 146 open reading frames (ORFs), while the Palpatine genome is 75,809 bp long with 140 ORFs. The gene synteny of both mycobacteriophages closely matches other phage genomes in subcluster E.

## ANNOUNCEMENT

The discovery and characterization of bacteriophages infecting the *Mycobacterium* genus advance the development of potential phage-based therapies, especially in combating multidrug-resistant pathogenic strains within the *Mycobacterium* genus (1). Here, we report the annotated genomes for two novel mycobacteriophages, Policronamos and Palpatine.

Policronamos and Palpatine were both isolated from compost collected in Provo, Utah (Table 1). Each sample was suspended in 7H9 broth and filtered through a 0.22 µm filter. Plaque assays of the filtrate were performed with host bacterium (*Mycobacterium smegmatis* mc^2^155) amalgamated in 7H9 broth, mixed with top agar, and plated onto 7H10 agar, then aerobically incubated at 37°C for 2 days. Three cycles of purification were performed to ensure isolation of individual phages, yielding Policronamos and Palpatine. Plaques for Policronamos were clear and circular (~5 mm in diameter), while those for Palpatine were turbid (~1.4 cm in diameter), potentially indicating lysogeny. High-titer lysates (>1 × 10^9^ PFU/mL) were prepared by flooding nearly confluent "web-plates" with Middlebrook 7H9 broth, incubating for 2 hours at room temperature, decanting, and filtering through a 0.22 µm filter. DNA was extracted from the lysate by using the Norgen Phage DNA Isolation Kit following the manufacturer’s protocol prior to library preparation and sequencing. Briefly, 0.5 µg of DNA per sample was used for DNA library preparation using the NEBNext Ultra DNA Library Prep Kit for Illumina (NEB, USA) following manufacturer’s recommendations and unique index codes were added to each sample. The DNA samples were sonicated to a size of 350 bp, then DNA fragments were end-polished, A-tailed, and ligated with the full-length adaptor for Illumina sequencing with further PCR amplification. PCR products were purified (AMPure XP system) and libraries were analyzed for size distribution with an Agilent 2100 Bioanalyzer and quantified using real-time PCR. The whole genomes were sequenced using Illumina NovaSeq X PE150 with the corresponding sequencing kit, generating 2.5–3.5 million 150 bp paired-end reads per genome. Reads were trimmed using TrimGalore v0.6.6 (2) with a minimum length of 20 bases and minimum phred quality score of 20. Trimmed reads underwent assembly with Newbler v.2.9 (3), prior to validation using CONSED v.2.9 (4) with default parameters.

**TABLE 1:** Features of the two mycobacteriophages belonging to subcluster E

Feature	Value for:	
	Policronamos	Palpatine
GPS coordinates	40.273539°N, 111.640718°W	40.273539°N, 111.640718°W
Isolation details (year, temperature, depth)	2023, 37°C, surface	2023, 35°C, surface
Sequence reads (millions)	1.41	1.10
Sequence depth (x coverage)	2,791	2,185
Genome length (base pairs)	75,894	75,809
GC content (%)	63%	63%
No. of ORFs	145	143
No. of orphams	6	0
No. of tRNAs	2	2
No. of ORFs with putative function (%)	47 (32%)	45 (31%)
No. of ORFs with no putative function, including orphams (%)	98 (68%)	98 (68%)

**Fig 1:**
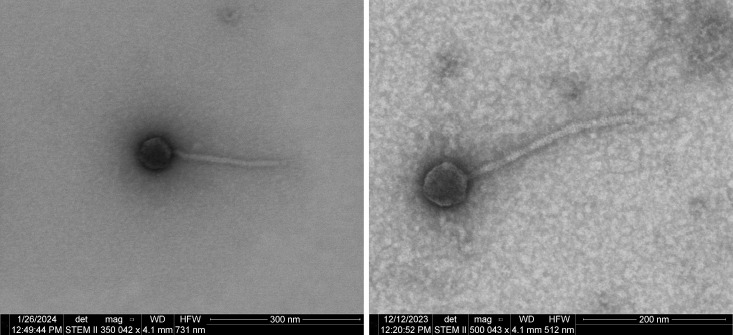
STEM images of Policronamos (left) and Palpatine (right) with 20 kV and an 86 pA probe. Micrographs were collected using a STEM II detector and an ion beam with a brightfield light in immersion mode at ~350,000x (Policronamos) or 500,000x (Palpatine) magnification on a Helios Nanolab 600 FEI instrument.

FASTA files of each genome sequence were first auto-annotated by the GeneMark 3.0 (5) and Glimmer 2.5 (6) algorithms within DNA Master v.5.23.6 (7) with default parameters. Automated gene annotations were manually verified using default parameters for: Starterator server v.1.2, web-based Phamerator v.600 genome maps (8), tRNAscanSE v.2.0 tRNA analysis (9), the HHPRED web server (10), GeneMarkS v.4.28 coding potential maps (11), PhageScope v.1.0 (12), and the NCBI BLASTP server (13). Negative-stained scanning transmission electron microscopy (STEM; 2% uranyl acetate) imaging showed that these phages have a siphovirus-like morphotype (Fig. 1).

BLASTN sequence similarity scores from the NCBI server were used to assign these phages to subcluster E. Policronamos contains six putative orphams (Table 1). We also identified frameshifts in both bacteriophages within their first Tail Assembly Chaperone open reading frame (ORF). The ongoing discovery and annotation of phages will augment our understanding of the diverse sequences and functions that exist in these microorganisms.

## Data Availability

The consensus genome sequences are available in GenBank with accession numbers PV165875 and PV105553 and in the Sequence Read Archive (SRA) with accession numbers SRX28150559 and SRX28150558 for Policronamos and Palpatine, respectively.
